# Clinical efficacy of chemotherapy in colorectal cancer patients over 80 years old

**DOI:** 10.1007/s00384-022-04222-7

**Published:** 2022-07-20

**Authors:** Dongdong Zhang, Xue Wang, Mingbao Zhang, Yafei Yin, Jianqiang Guo

**Affiliations:** 1 Department of Gastroenterology, The Second Hospital, Cheeloo College of Medicine, Shandong University, 247 Beiyuan Street, Jinan, Shandong People’s Republic of China; 2 Jinan Maternal and Child Health Hospital, Jinan, 250001 Shandong China

**Keywords:** Colorectal cancer, Chemotherapy, Survival analysis, Super elderly patients

## Abstract

**Purpose:**

Colorectal cancer (CRC) is a common and aggressive gastrointestinal cancer, and the prognostic impact associated with chemotherapy in super elderly (over 80 years old) patients remains poorly defined. We aimed to define the effect of chemotherapy on the prognosis of patients with CRC over 80 years old.

**Patients and methods:**

A retrospective study including CRC patients over 80 years old was conducted. The patients were screened from the Surveillance Epidemiology and End Results (SEER) database from 2010 to 2015. Overall survival (OS) and cancer-specific survival (CSS) were applied as the primary and secondary outcome. Cox proportional hazards regression models were used to evaluate factors associated with OS and CSS. Survival curves of OS and CSS were estimated by Kaplan–Meier method and compared by log-rank test.

**Results:**

In total, 14,748 CRC patients over 80 years old were included in this study. The median patient age was 85 (IQR: 82–87). All patients were divided into surgical group and non-surgical group. The OS and CSS of the surgical group were significantly better than those of the non-surgical group (*P* < 0.001). Chemotherapy can improve OS and CSS for patients with stage III and IV (*P* < 0.001) in surgical group. For the super elderly patients with CRC, chemotherapy significantly improved OS and CSS in all TNM stages in non-surgical group.

**Conclusion:**

For super elderly patients with colorectal cancer, tumor treatment should not be abandoned because of their age. It is necessary to carry out clinical trials in super elderly patients.

## Introduction

Colorectal cancer (CRC) is one of the most common cancers worldwide. According to data from cancer statistics, CRC ranks third in morbidity and second in mortality [1]. In addition to incidence rate, the age of onset is gradually improving. Among CRC patients in the US in 2017, 27% of new cases and 40% of deaths in women occurred in people aged 80 and over, compared with 18% and 27% in men, respectively [2]. For patients with CRC, surgery and chemotherapy are considered the preferred treatments, which significantly reduces the mortality and improves the prognosis [3, 4]. For the super elderly patients (over 80 years old), due to the comorbidity and frailty issues, there are some confusions in the choice of treatment for CRC. Different from patients in early age onset, elderly patients should consider receiving active treatment after risk assessment. In previous studies, there were many studies on the surgical methods and chemotherapy regimens of CRC patients, but few studies have shown the impact of two treatment methods, especially chemotherapy, on the prognosis of super elderly patients [5–8]. There is no representative randomized controlled study on the antitumor treatment of CRC in super elderly patients. More importantly, even super elderly patients actively choose because of their health status, so strong selection bias needs to be considered. Due to the lack of real-world research data, the medical community currently lacks guiding opinions on super elderly patients. The huge heterogeneity of health status in super elderly patients with CRC (due to multiple comorbidities) makes it difficult to establish randomized controlled trials in these populations. The current clinical trial design may not be suitable for super elderly patients. Moreover, for CRC patients with different stages, the effects of chemotherapy and surgery are also different [9, 10]. Therefore, in the absence of clinical trial research, the purpose of this retrospective study was to investigate the efficacy of chemotherapy in super-elder patients with CRC in different TNM stages.

## Material and methods

### Data source

All patients were selected from the Surveillance Epidemiology and End Results (SEER) database using SEER*Stat software (www.seer.cancer.gov/seerstat) version 8.3.9.1. The SEER database provided clinical, surgery, and survival information of cancer patients from 18 cancer registries from the USA, which account for approximately 30% population of the USA.

### Patient selection

We selected patients who were diagnosed CRC from 2010 to 2015 in this study. The selection criteria included the following: (1) patients who were over 80 years old; (2) ICD-O-3 site codes: ‘Colon and Rectum’; (3) ICD-O-3 behavior codes: ‘Malignant’; (4) complete information of demographic (age, sex, race), clinical (location, size, number, TNM stage), pathologic (histologic type, grade), and therapy (surgery, chemotherapy) information. The exclusion criteria were in the following: (1) incomplete above information; (2) more than one primary cancers; and (3) diagnosed at autopsy. (4) The patient died within 1 month after surgery.

### Statistical analysis

The following demographic and pathological variables were included in this study: gender (male and female), race (white, black, and others), location (left, transverse, right), cancer-specific death (yes or no), survival (survival time), TNM stage (by American Joint Committee system), surgery for primary site (yes or no), chemotherapy (yes or no), carcinoembryonic antigen (CEA) level (normal or elevated), histology (adenocarcinoma, mucinous adenocarcinoma, signet ring cell carcinoma). The SEER database is a public database, in which all data are anonymized, and no personal information is involved in the use of data. Therefore, this study does not need the approval of ethics committee and informed consent of patients. The data from the SEER database was only used for research.

Overall survival (OS) was the primary outcome of this study, and the cancer-specific survival (CSS) was the second outcome. OS was defined as the time from diagnosis to death by all kinds of reasons. CSS was defined as the time from diagnosis to death by CRC. The patients were divided into two groups for statistical analysis according to surgery treatment or not. We then used cox proportional hazards regression models to evaluate factors associated with OS for each group. Subsequently, each group was divided into four subgroups according to TNM stage to study the effects of chemotherapy on OS and CSS. According to the chemotherapy record of SEER database, survival analysis was performed in each subgroup according to whether chemotherapy was implemented. Survival curves were estimated by Kaplan–Meier method and compared using the log-rank test. Statistical analyses were performed with software programs (R software, version 4.1.1). All tests were two sided, and *P* < 0.05 was considered statistically significant.

## Results

### Demographic and clinicopathological characteristics

According to the inclusion and exclusion criteria, a total of 14,748 patients were selected for this study, who were diagnosed with CRC over 80 years old (Table 1). The median age of these patients was 85 (IQR: 82–87). 96.4% of the patients received surgical treatment, and 3.6% of the patients did not receive surgical treatment. Most patients were of white race (84.5%), and the tumor was located in the left (48.5%), transverse (18.2%), and right (33.3%) colon. In total, 70.7% of all patients were moderately differentiated. For all patients, only 18.1% of them received chemotherapy; in addition, 48.7% of the patients in the nonoperative group received chemotherapy, and 17.0% of the patients in the operative group received chemotherapy. In the final pathological report, most patients (88.8%) were adenocarcinoma, and the 9.8% of the patients were mucinous adenocarcinoma.

**Table 1:** Baseline characteristics of CRC aged over 80

	Total (*n*, %)	Nonoperative group (*n*, %)	Operative group (*n*, %)
Total	14,748 (100%)	526 (3.60%)	14,222 (96.4%)
Sex			
Male	6338 (43.0%)	257( 48.9%)	6081 (42.8%)
Female	8410 (57.0%)	269 (51.1%)	8141 (57.2%)
Race			
White	12,457 (84.5%)	435 (82.7%)	12,022 (84.5%)
Black	1040 (7.10%)	49 (9.30%)	991 (7.00%)
Others	1251 (8.50%)	42 (8.00%)	1209 (8.50%)
Location			
Left	7154 (48.5%)	93 (17.7%)	7061 (49.6%)
Transverse	2678 (18.2%)	35 (6.70%)	2643 (18.6%)
Right	4916 (3.33%)	398 (75.7%)	4518 (31.8%)
Grade			
Well differentiated	1017 (6.90%)	47 (8.9%)	970 (6.8%)
Moderately differentiated	10,432 (70.7%)	385 (73.2%)	10,047 (70.6%)
Poorly differentiated	3299 (22.4%)	94 (17.9%)	3205 (22.5%)
TNM stage			
I	3170 (21.5%)	124 (23.6%)	3046 (21.4%)
II	5217 (35.4%)	109 (20.7%)	5108 (35.9%)
III	4699 (31.9%)	92 (17.5%)	4607 (32.4%)
IV	1662 (11.3%)	201 (38.2%)	1461(10.3%)
CEA			
Normal	8182 (55.5%)	207 (39.4%)	7975 (56.1%)
Elevated	6566 (44.5%)	319 (60.6%)	6247 (43.9%)
Chemotherapy			
No	12,080 (81.9%)	270 51.3%)	11,810(83.0%)
Yes	2668 (18.1%)	256(48.7%)	2412(17.0%)
Histology			
Adenocarcinoma	13,097 (88.8%)	501 (95.2%)	12,596 (88.7%)
Mucinous adenocarcinoma	1451 (9.80%)	19 (3.60%)	1432 (10.1%)
Signet ring cell carcinoma	179 (1.20%)	6 (1.10%)	173 (1.20%)

The result of multivariate Cox regression analysis is shown in Table 2. The multivariate analysis indicated that sex, chemotherapy, and surgery were protect factors for OS in super elder group (HR:0.82, 0.55, 0.39, respectively, *P* < 0.001).

**Table 2:** Results of multivariate Cox regression model showing the association of variables with overall survival

	HR (95%CI)	*P* value
Sex		
Male	Ref	
Female	0.82 (0.78–0.85)	< 0.001
Race		
White	Ref	
Black	1.07 (0.99–1.16)	0.084
Others	0.87 (0.81–0.94)	< 0.001
Location		
Left	Ref	
Transverse	1.03 (0.98–1.09)	0.294
Right	1.04 (0.99–1.09)	0.089
Grade		
Well differentiated	Ref	
Moderately differentiated	1.03 (0.94–1.12)	0.535
Poorly differentiated	1.23 (1.12–1.35)	< 0.001
TNM stage		
I	Ref	
II	1.04 (0.99–1.10)	0.206
III	1.83 (1.72–1.95)	< 0.001
IV	5.12 (4.73–5.54)	< 0.001
CEA		
Normal	Ref	
Elevated	1.40 (1.35–1.47)	< 0.001
Chemotherapy		
No	Ref	
Yes	0.55 (0.52–0.58)	< 0.001
Histology		
Adenocarcinoma	Ref	
Mucinous adenocarcinoma	1.09 (1.02–1.17)	0.011
Signet ring cell carcinoma	1.11 (0.93–1.32)	0.236
Surgery		
No	Ref	
Yes	0.39 (0.35–0.43)	< 0.001

### Kaplan–Meier survival analysis

**Fig. 1:**
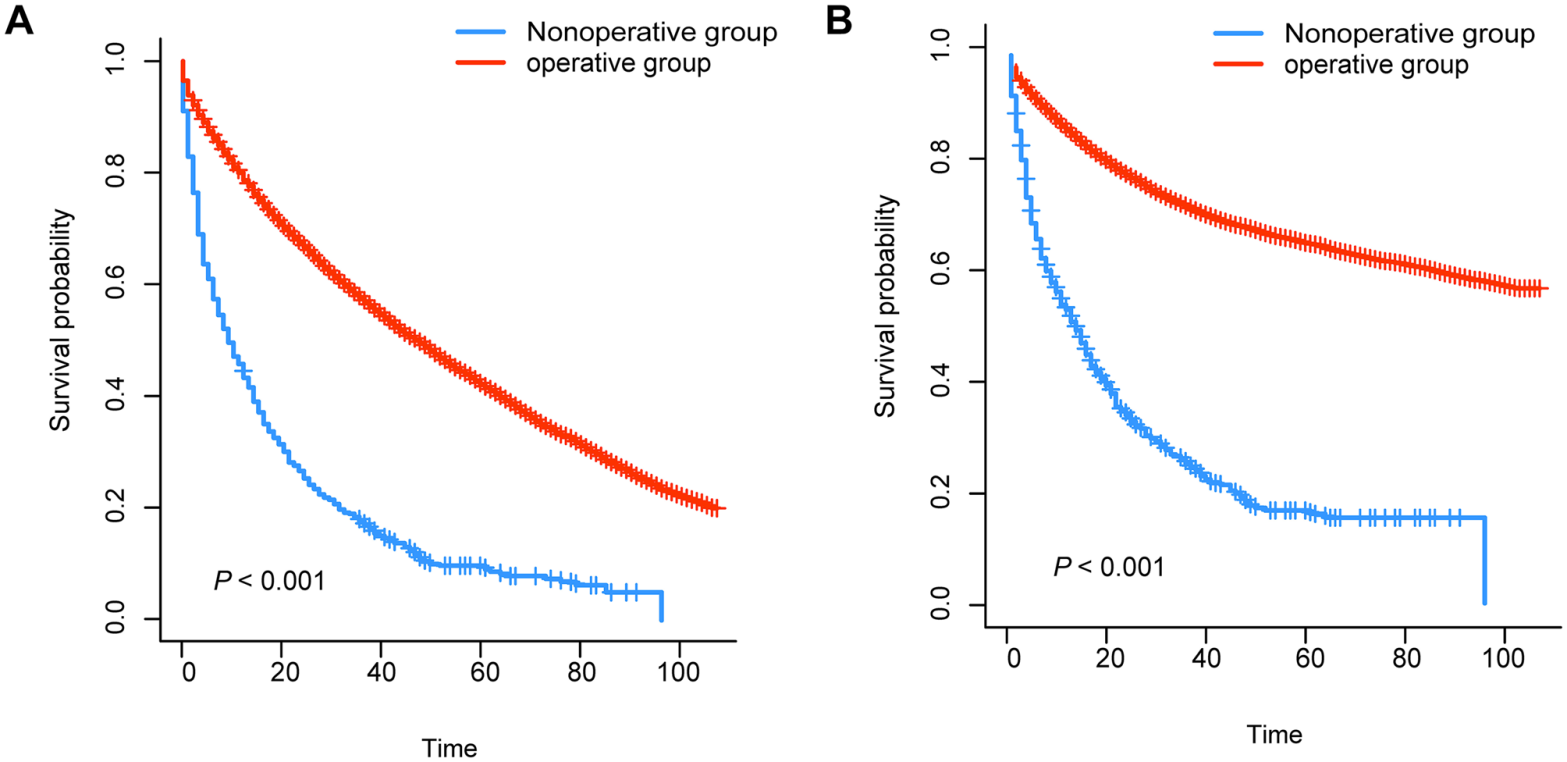
Kaplan–Meier plots of OS (**A**) and CSS (**B**) for super elderly patients in surgical group and non-surgical group

**Fig. 2:**
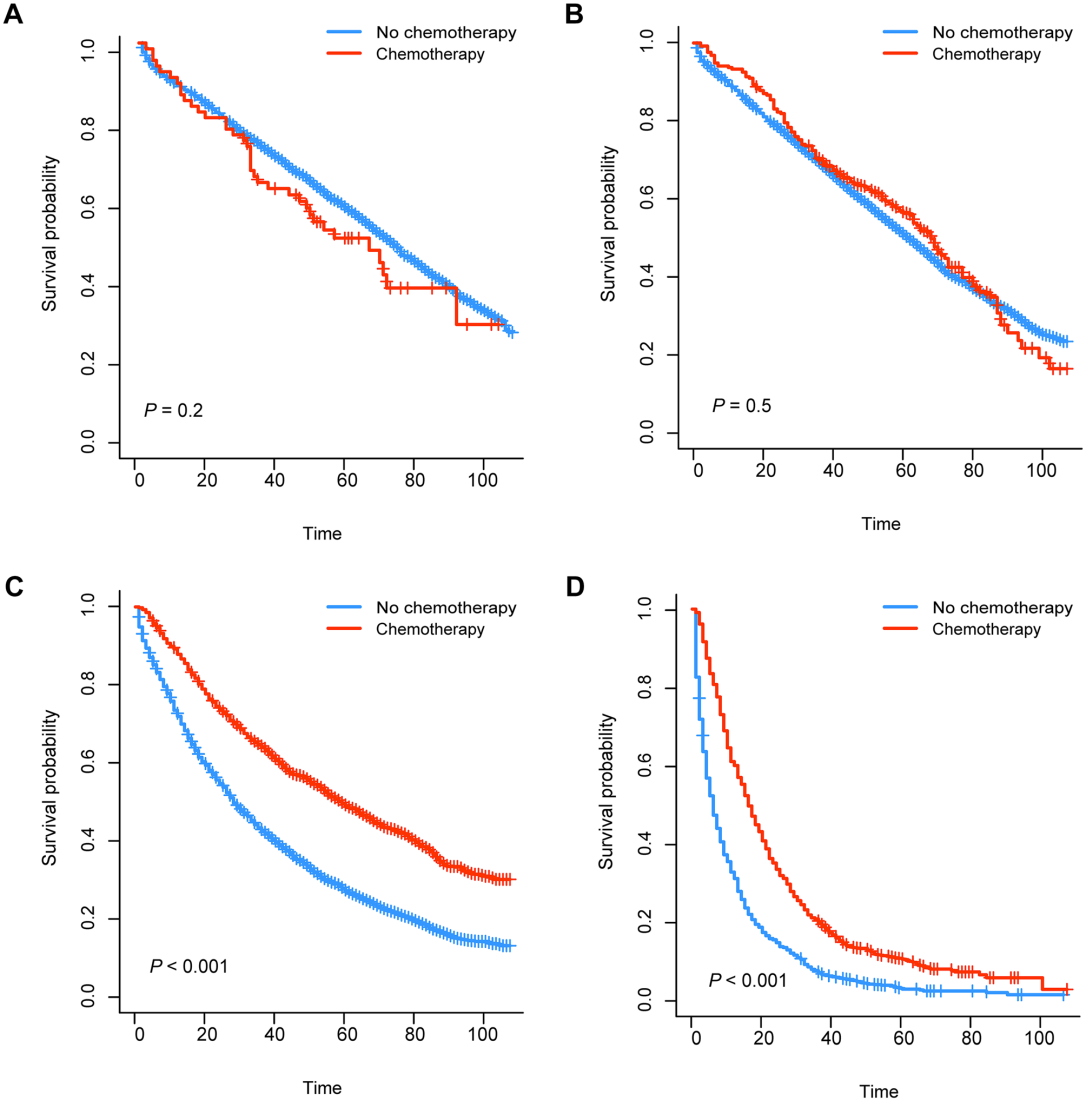
Kaplan–Meier plots of OS for super elderly patients in surgical group in stage I (**A**), stage II (**B**), stage III (**C**), and stage IV (**D**)

**Fig. 3:**
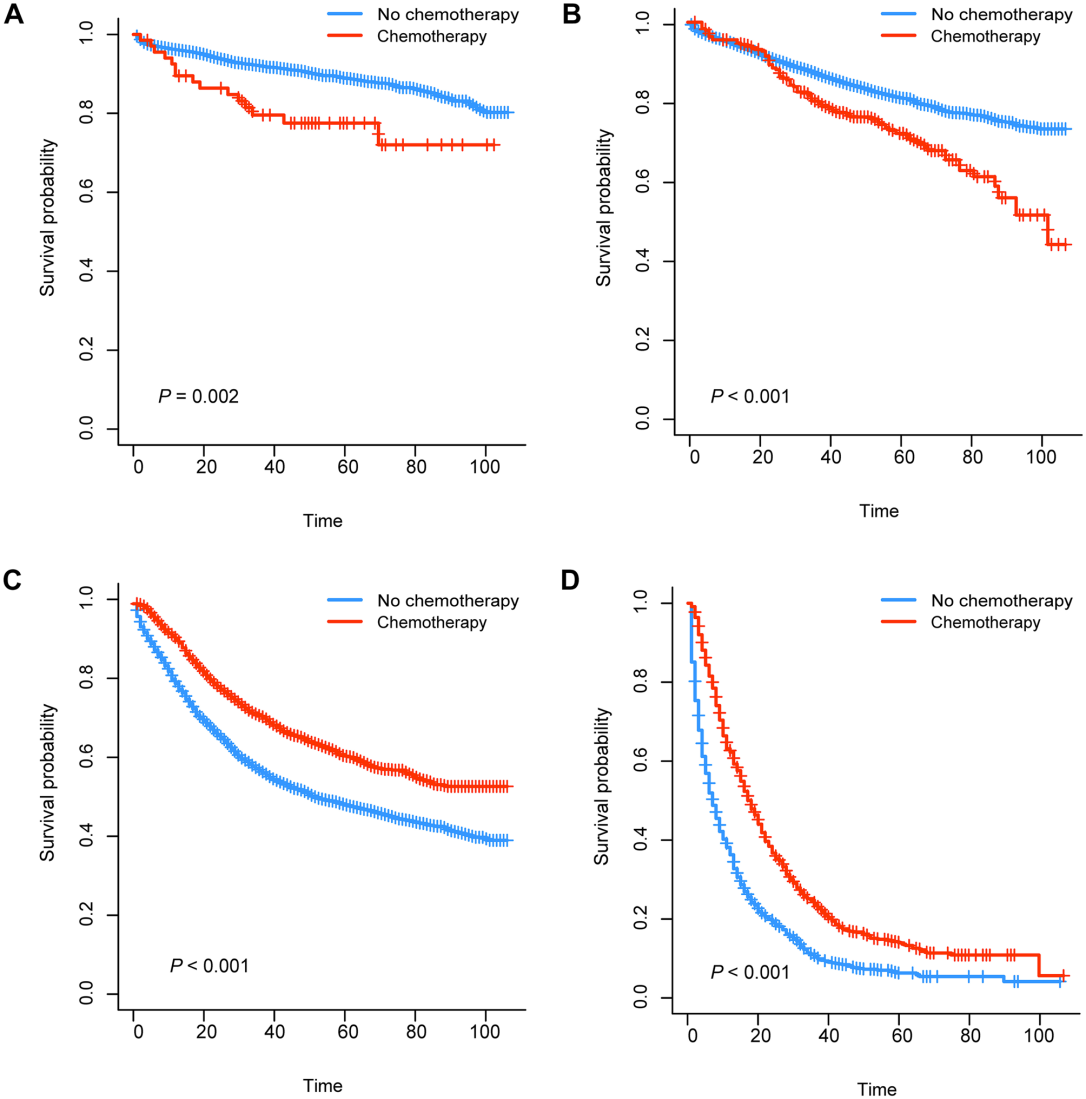
Kaplan–Meier plots of CSS for super elderly patients in surgical group in stage I (**A**), stage II (**B**), stage III (**C**), and stage IV (**D**)

**Fig. 4:**
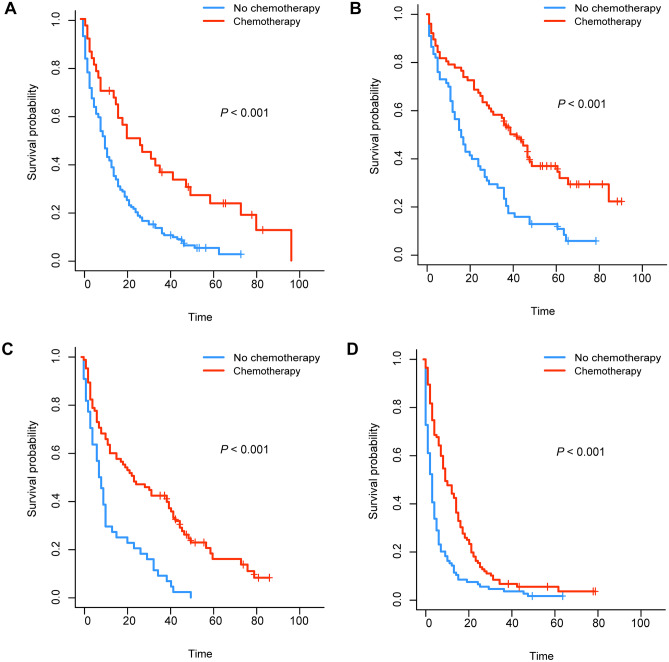
Kaplan–Meier plots of OS for super elderly patients in non-surgical group in stage I (**A**), stage II (**B**), stage III (**C**), and stage IV (**D**)

**Fig. 5:**
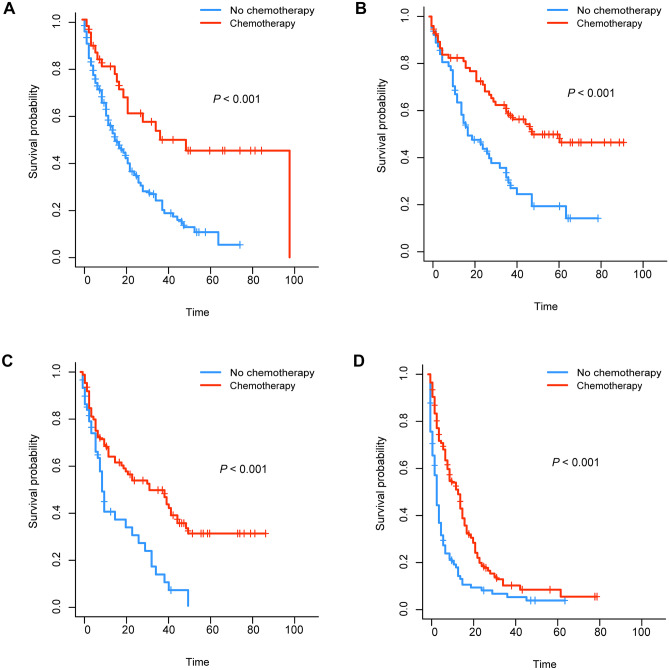
Kaplan–Meier plots of CSS for super elderly patients in non-surgical group in stage I (**A**), stage II (**B**), stage III (**C**), and stage IV (**D**)

We generated K-M survival curves with regard to the prognosis of patients in the surgical group and non-surgical group. For the super elderly patients with CRC, whether OS or CSS, the prognosis of the surgical group was significantly better (*P* < 0.001) than that of the non-surgical group (Fig. 1). As shown in Fig. 2, in surgical group, OS of patients in stage III and stage IV receiving chemotherapy were higher than those in patients not receiving chemotherapy. On the other hand, CSS of patients in stage III and stage IV was also higher than that in patients not receiving chemotherapy (Fig. 3). For the super elderly CRC patients in non-surgical group, chemotherapy significantly improved OS (Fig. 4) and CSS (Fig. 5) in all TNM stages.

## Discussion

In the present study, we analyzed the effects of chemotherapy on OS and CSS in super elderly patients in different TNM stages. With the global aging process, there have been many studies on the prognostic factors of super elderly patients with CRC, but these studies generally define the elderly as over 65 years old. For patients over 80 years old, relevant research is also important. Because of the special situation of super elderly patients, there are few studies on the prognostic factors of CRC over 80 years old [11]. The special manifestations of super elderly patients are as follows: (1) They are often complicated with a variety of basic diseases, which has obvious obstacles to surgery and chemotherapy; (2) liver and kidney function and hematopoietic system function decline and cannot tolerate radical treatment schemes; (3) the expected survival time is short, and it is difficult to predict the future for the treatment completed at risk. Therefore, the choice of treatment for super elderly patients with CRC is often a dilemma. To the best of our knowledge, this study is the first to analyze the effects of chemotherapy on OS and CSS in super elderly patients in different TNM stages.

First of all, it is believed that for elderly patients with CRC(> 75 years), surgery treatment has an acceptable survival rate, and age should not be the reason for refusing surgery [12]. In our study, it revealed that surgery can significantly improve the prognosis of the super elderly CRC patients. Whether OS or CSS, the prognosis of patients in the operative group is better than that in the nonoperative group (*P* < 0.001). Therefore, based on the results of this paper, surgical treatment is recommended for super elderly patients with CRC. However, physical and nutritional status need to be fully evaluated before operation [13, 14]. Improving the surgical selection rate of elderly patients may improve the long-term prognosis, but it may be at the cost of higher 30-day mortality [15].

Second, chemotherapy plays an important role in CRC treatment. Patients with CRC after operation were divided into four groups according to TNM stage, and the effects of chemotherapy on each stage were observed. We found that some stage I super elderly patients with CRC received chemotherapy. The available data from the SEER database were lack of reasons of chemotherapy for post-surgery CRC patients, though it was well known that adjuvant therapy after surgery was not recommended in the standard therapeutic method according the NCCN guidelines and other guideline for these patients [13, 16]. Due to the lack of original records of chemotherapy, we do not know the exact reason for chemotherapy in these patients. Therefore, we can only speculate through the existing data. There may be many reasons, including positive margins, lymphovascular invasion, or SM3 invasion (submucosal invasion to the lower third of the submucosal level); at the same time, the patient refuses to operate again. Other possible causes are postoperative recurrence or nonstandard treatment. In order to figure out this problem, we may need larger sample size data and relevant research specifically for postoperative chemotherapy of stage I colon cancer, as well as detailed data on the causes of chemotherapy. There is controversy in the previous literature on patients with stage II. It is generally accepted that high-risk patients are recommended for chemotherapy. In one review article, patients with pT4, lymphovascular invasion, perineural invasion, obstruction, perforation, or high risk in gene expression signature in stage II or in stage III, should accept chemotherapy [17]. For patients with stage I and stage II CRC, chemotherapy could not improve OS (*P* > 0.05). Therefore, for super elderly CRC patients accepted surgical treatment, stage I and stage II patients who are not recommended for chemotherapy. Due to the limitations of the database, the relevant risk factors of stage II mentioned in the above literature cannot be analyzed.

It was found that the CSS of patients with chemotherapy in stage I/II surgical group was lower than that of patients without chemotherapy. To clarify this question, we analyzed the Cox proportional hazard regression model for the two groups of super elderly CRC patients. The *P* values of chemotherapy factor in the two groups were 0.303 and 0.160 respectively, and the *P* values of log-rank test were 0.002 and < 0.001 respectively. Then, we performed the same analysis on all remaining groups, and both were consistent. It is known that log-rank test belongs to nonparametric method, and Cox belongs to semi parametric method. When the conditions are met, the efficiency of parametric test is higher than that of nonparametric test, and Cox can prevail. Secondly, there may be a bias between chemotherapy patients and non-chemotherapy patients. Patients who choose chemotherapy may have more serious conditions, although we did not find significant differences in the included factors. If we want to understand this puzzle, we may need a larger sample size and prospective research. Finally, given that OS is widely recognized in cancer research, although the CSS results are equivocal, it can also be concluded from the results of OS that stage I/II super elderly CRC patients cannot benefit from chemotherapy.

For stage III and IV CRC, this study indicates chemotherapy can improve OS and CSS in super elderly patients (*P* < 0.001). A study that included four randomized, controlled trials suggested that patients with stage III CRC of all ages should be treated with XELOX or FOLFOX as standard treatment option [18]. For stage III and IV CRC patients, the median progression-free survival (PFS) of patients receiving chemotherapy was significantly longer than that of patients not receiving chemotherapy (the mean age was 61.3 years, *P* < 0.001) [19]. Unfortunately, there is no data on specific chemotherapy regimen in SEER database, so correlation analysis cannot be carried out. However, through previous studies, it can be seen that for super elderly CRC patients, it is also very important to choose an appropriate chemotherapy regimen, and further clinical research is also needed.

OS and CSS were improved in all TNM stages after chemotherapy in nonoperative group (P < 0.001). A phase II clinical study involving 40 patients (median age 77.3 years) suggested that 5-fluorouracil and irinotecan (FOLFIRI 1) regimen was an effective treatment for elderly subjects with advanced CRC in good clinical condition [20]. In addition, in a phase III trial of elderly patients with metastatic CRC (media age was 80 years), 71 patients were randomly divided into LV5FU2 group, 71 into simplified LV5FU2 group, 70 into LV5FU2 irinotecan group, and 70 into FOLFIRI group. It is considered that irinotecan cannot significantly improve the progression-free survival (PFS) or overall survival (OS) of patients. At the same time, compared with simplified LV5FU2, the classic LV5FU2 regimen can improve the OS of patients [21]. An open label randomized trial involving 459 patients with advanced CRC showed that frail and elderly patients could participate in randomized controlled trials through appropriate adjustments, including reducing the starting dose of chemotherapy. Overall, combination drugs including oxaliplatin were superior to single agent fluoropyrimidine, although the primary endpoint of PFS was not reached. Capecitabine did not significantly improve the quality of life of patients with CRC compared with fluorouracil [22]. Therefore, super elderly patients with colorectal cancer, especially those without surgical treatment, should not refuse chemotherapy due to age.

However, there are still many limitations in this study. First, the data studied in this paper come from a single database, Multi center research is more meaningful for the treatment choice of super elderly CRC patients. Secondly, SEER database only has the data of chemotherapy or not, but there is no specific data for the chemotherapy regimen. For the elderly CRC patients over 80 years old, the patient’s own combined diseases and the choice of chemotherapy regimen (chemotherapy drugs and dose) have a great impact on the prognosis. If these two data are recorded, it should be convincing for this research. Third, there is less data for patients in nonoperative group. Therefore, multicenter prospective research is needed.

## Conclusion

For super elderly patients with CRC, surgery can improve OS and CSS. Chemotherapy can improve OS and CSS in patients with stage III and IV CRC, but not in patients with stage I and II CRC. For patients without surgical treatment for various reasons, chemotherapy can improve OS and CSS for patients in all TNM stages. For these patients, age should not be the only indicator to decide whether to operate or chemotherapy. Patients with different TNM stages should adopt different treatment schemes. In addition, despite the difficulties in implementation, clinical trials in super elderly patients should be considered.
